# Transition Boundary from Laminar to Turbulent Flow of Microencapsulated Phase Change Material Slurry—Experimental Results

**DOI:** 10.3390/ma17246041

**Published:** 2024-12-10

**Authors:** Krzysztof Dutkowski, Marcin Kruzel, Martyna Kochanowska

**Affiliations:** Department of Mechanical and Power Engineering, Koszalin University of Technology, Raclawicka Street 15-17, 75-620 Koszalin, Poland; krzysztof.dutkowski@tu.koszalin.pl (K.D.); kochanowska34@gmail.com (M.K.)

**Keywords:** critical Reynolds number, microencapsulated PCM slurry, experimental investigation

## Abstract

An ice slurry or an emulsion of a phase change material (PCM) is a multiphase working fluid from the so-called Latent Functional Thermal Fluid (LFTF) group. LFTF is a fluid that uses, in addition to specific heat, the specific enthalpy of the phase change of its components to transfer heat. Another fluid type has joined the LFTF group: a slurry of encapsulated phase change material (PCM). Technological progress has made it possible for the phase change material to be enclosed in a capsule of the size of the order of micrometers (microencapsulated PCM—mPCM) or nanometers (nanoencapsulated PCM—nPCM). This paper describes a method for determining the Reynolds number (Re) at which the nature of the flow of the mPCM slurry inside a straight pipe changes. In addition, the study results of the effect of the concentration of mPCM in the slurry and the state of the PCM inside the microcapsule on the value of the critical Reynolds number (Re_cr_) are presented. The aqueous slurry of mPCM with a concentration from 4.30% to 17.20% wt. flowed through a channel with an internal diameter of d = 4 mm with a flow rate of up to 110 kg/h (Re = 11,250). The main peak melting temperature of the microencapsulated paraffin wax used in the experiments was around 24 °C. The slurry temperature during the tests was maintained at a constant level. It was 7 °C, 24 °C and 44 °C (the PCM in the microcapsule was, respectively, a solid, underwent a phase change and was a liquid). The experimental studies clearly show that the concentration of microcapsules in the slurry and the state of the PCM in the microcapsule affect the critical Reynolds number. The higher the concentration of microcapsules in the slurry, the more difficult it was to maintain laminar fluid flow inside the channel. Furthermore, the laminar flow of the slurry terminated at a lower critical Reynolds number when the PCM in the microcapsule was solid. Caution is advised when choosing the relationship to calculate the flow resistance or heat transfer coefficients, because assuming that the flow motion changes at Re = 2300, as in the case of pure liquids, may be an incorrect assumption.

## 1. Introduction

A slurry of microencapsulated phase change material (mPCM) is another type of so-called Latent Functional Thermal Fluid (LFTF), along with ice slurries or PCM emulsions [1,2,3]. Using PCM as a capsule filling allows for transporting high-density thermal energy using, in addition to sensible heat, the heat of the phase change. It eliminates the disadvantages of using PCM as a direct additive to the base liquid, e.g., the potential clogging of pipes due to the merging of liquid PCM droplets or an increase in thermal resistance due to PCM sticking to the inner surface of the pipes. The thin coating of the microcapsule ensures the close contact of the PCM with the base liquid. As a result, the resistance to heat exchange between the substances is reduced, enabling rapid energy exchange [4,5]. The microscopic dimensions of the capsules give the PCM a large ratio of external surface area to occupied volume and the ability to disperse evenly in the liquid. An mPCM slurry can be used as a filling for thermal energy storage [6], as a circulating fluid in heat exchange systems [7,8] or as electronic cooling [9]. It is considered a promising fluid because the fluid, on a macroscopic scale, looks and behaves the same when the PCM in a microcapsule is solid and when the PCM is a liquid. It should be noted that the use of an mPCM slurry, on the one hand, results in improved heat transfer and a reduction in the required mass of the fluid circulating in the heat exchange system, and, on the other hand, it requires an increase in the energy input necessary for its pumping [10,11,12]. Therefore, heat transfer studies and evaluations of the benefits of using an mPCM slurry should not be carried out in isolation from the analysis of the increase in energy input necessary to force the slurry flow. Moreover, there is no information on the critical Reynolds number, i.e., the Reynolds number at which the nature of the flow changes from laminar to turbulent. The results of the authors’ preliminary studies indicate that caution is advised when choosing the relationship to calculate the flow resistance or heat transfer coefficients, because assuming that the flow motion changes at Re = 2300, as in the case of pure liquids, may be an incorrect assumption.

The paper by Zhang et al. [13] describes the results of thermal and flow tests of a tetrabutylammonium bromide (TBAB) clathrate hydrate slurry (CHS) in a plate heat exchanger and a double-tube heat exchanger. Thermal tests were carried out in the range of the turbulent flow of the slurry. They confirmed that the higher the TBAB concentration, the more intense the heat exchange and the more significant the pressure drop in the slurry flow. Also, in another study [14], the heat exchange coefficients and the pressure drop induced by a slurry flow were determined experimentally. The authors used poly-α-olefin (PAO) in their studies, to which indium nano-PCM surrounded by silica was added. The authors showed that with the increase in the concentration of nanocapsules in the slurry, at the same slurry flow rate, the measured flow resistances were several times higher than for the flow of the base liquid without the addition of nanocapsules. The increase in the flow resistance of the slurry resulting from the increase in both its flow rate and the concentration of microcapsules was also confirmed in research works [15,16,17,18]. The cited studies presented the pressure drop characteristics from the flow rate or the flow velocity of the slurry, so it was difficult to determine how wide a range of the Reynolds number the studies covered. Also, the shape of the characteristics did not allow for the assessment of whether the scope of the experiment included laminar flow, turbulent flow, or both cases. An exception is the work of Zhang et al. [19]. The pressure drop characteristics from the mass flow rate of the slurry indicate that the authors were able to carry out experimental studies of the flow of TBAB clathrate hydrate slurry in the range of laminar and turbulent motion. The presented characteristics show that the higher the TBAB concentration in the slurry, the higher the recorded pressure drop. Moreover, the change in the trend of the curves indicates that the higher the TBAB concentration in the slurry, the higher the mass flow rate, and a transition from laminar to turbulent motion occurred. However, based on the presented data, it is impossible to determine at what value of the Reynolds number the change, like the fluid motion, took place.

The pressure drop caused by flow resistance of micro- or nanoencapsulated mPCM slurry as a function of the Reynolds number was presented, among others, in research works [20,21,22,23]. However, due to the research conducted in the range of small Reynolds numbers (laminar flow), the works do not allow determining whether and to what extent the concentration of capsules in the slurry affects the critical Reynolds number. A precise assessment of the effect of microcapsules on the critical Reynolds number can be made based on the characteristics of the friction factor of the slurry from the Reynolds number. There are few works devoted to research in which, in addition to determining the heat transfer coefficients, the friction factor value was determined. However, the authors of the works focused mainly on assessing the possibility of calculating the linear loss coefficient in the slurry flow from the relationship proposed by Hagen–Poiseuille (for laminar flow) and the Blasius equation (for turbulent flow). The evaluation of the Reynolds number range at which these relations are valid for the flow of an mPCM slurry was not the subject of the publication. Yamagishi et al. [24] presented the effect of the Re number on the friction factor for water and an aqueous slurry of microencapsulated octadecane (C_18_H_38_). The particle volume fraction in the slurry was 15% and 30%. The slurry flowed inside a straight pipe with an internal diameter of 10.1 mm and a total length of 10.5 m. The calculated values of the pressure drop over a length of 8 m showed that the friction factor could be calculated from the Hagen–Poiseuille and Blasius relations for laminar and turbulent slurry flow, respectively. The transition boundary from laminar to turbulent movement occurred in the Re number range from 2000 to 3000.

Ho et al. [25,26] measured the flow resistance of a microencapsulated PCM slurry through a minichannel heat sink. The concentration of microcapsules in water was 2%, 5% and 10%, and the flow was in the Re = 133 ÷ 1515 range. It was shown that the results of Fanning friction factor calculations follow the trend line for laminar flow up to the value of Re < 1000. After exceeding this value of the Reynolds number, the experimental results indicate a change in the trend of the characteristics. This may indicate an earlier change, such as fluid movement during the tests. The concentration of microcapsules did not affect the value of the critical Reynolds number. The tests described in [27] were also carried out in the laminar flow range of the mPCM slurry (Re < 3000). An aqueous slurry of n-octadecane microcapsules, with a mass concentration of up to 20%, flowed inside the heat exchanger minichannels. When the concentration of microcapsules in the slurry was up to 10%, the slurry behaved like a Newtonian fluid, and the friction factor could be calculated from the Hagen–Poiseuille relationship. A change like fluid motion was observed when Re ≈ 2000. The greater the concentration of microcapsules in the slurry, the more the boundary at which the fluid motion-like change occurred disappeared. The authors explained this fact by the damping effect of microcapsules on the occurrence of transitional flow and turbulent structure.

The studies of the Fanning friction factor in the range of Re < 7000 carried out by Inaba et al. [28,29] indicate that the concentration of microcapsules may affect the critical Reynolds number. During the studies, a slurry containing 20% of small PCM microcapsules (n-tetradecane in a 1.5 μm microcapsule) was used, to which up to 50% of large microcapsules (n-decosane in a 17 μm microcapsule) were added. The mPCM slurry without adding large mPCM showed the characteristics of a non-Newtonian fluid. The higher the concentration of large microcapsules in the slurry, the more its behaviour began to resemble the flow of a Newtonian fluid, the friction factor of which could be calculated from the Blasius relationship in turbulent motion. At that time, the friction factor characteristic of Re resembled the characteristic of a Newtonian fluid with a clear transition boundary from laminar to turbulent flow. The higher the concentration of large microcapsules in the slurry, the more visible the critical Reynolds number was, and its value decreased, tending to Re ≈ 2000. The authors explained the Newtonian behaviour of the slurry, despite the addition of increasing amounts of large microcapsules, by the Thoma effect, which consists of the suppression of turbulence by large structures contained in the slurry. The influence of the microcapsule concentration on the critical Reynolds number was considered in [30]. A tetra-butylammonium bromide (TBAB) clathrate hydrate slurry (CHS) with a volume fraction of 0–20.0 vol% flowed through a tube with an internal diameter of 6 mm or 14 mm. The 3 m long pipe was divided into two equal parts. The first was the hydraulic stabilization zone. The second one measured the pressure drop in the slurry flow. The authors found that the transition boundary from laminar to turbulent motion depended on the clathrate hydrate concentration and ranged from Re = 1500 to 2800 (for a 6 mm diameter tube) or Re = 1400 to 2100 (for a 14 mm diameter tube). The higher the clathrate hydrate concentration, the more difficult it was to obtain turbulent fluid flow, which, analogously to previous studies, the authors explained by the suppression of turbulent liquid vortices by solid particles.

The literature review shows that there are no articles that directly address the issue of the effect of the concentration of a slurry of micro- or nano-encapsulated PCM, and especially the state of matter of the PCM, on the Reynolds number, at which the nature of the fluid flow changes. Uncritical use of the relationship to calculate the flow resistance or heat transfer coefficients, assuming that the change, like fluid movement, occurs analogously to the flow of a “pure” liquid, may be an incorrect assumption. This article presents a method for determining the critical Reynolds number (Re_cr_) at which the nature of the flow of an aqueous slurry of micro-encapsulated phase change material (mPCM) changes from laminar to turbulent. The method is based on determining the Poiseuille number (Po), which is a constant value for laminar fluid flow. The tests used an aqueous slurry of microcapsules with mass concentrations of 4.30%, 6.45%, 8.60%, 10.75%, 12.90%, 15.05% and 17.20%, respectively. The mass flow rate of the slurry was increased stepwise, up to 110 kg/h, which corresponded to the Reynolds number Re = 11,250. The slurry flowed inside a straight section of a pipe with an internal diameter of d = 4 mm. The slurry temperature during the tests was constant and amounted to 7 °C, 24 °C and 44 °C. Such a value of the slurry temperature caused the PCM in the microcapsule to become a solid, undergo a phase change and become a liquid. The influence of the state of the PCM and the concentration of mPCM in the slurry on the critical Reynolds number was determined. It was shown that the higher the concentration of microcapsules in the slurry, the lower the Re_cr_ value, and a transition from laminar flow to turbulent flow occurred. Moreover, it was shown that when the PCM in the microcapsule was a solid, the laminar flow of the slurry was more challenging to maintain. On the contrary, when the PCM in the microcapsule was a liquid, a higher Re_cr_ value was achieved before the change, like fluid movement, occurred.

## 2. Materials and Methods

The slurry samples used for the study were prepared by mixing together, in the appropriate proportions, water and a commercial product from Microtek Labs (Moraine, OH, USA) called MICRONAL^®^ 5428 X. This was an aqueous concentrate of microencapsulated paraffin. The microcapsules were made of polymethyl methacrylate polymer, and their size was 1–5 μm. The proportion of microcapsules in the concentrate was 43 ± 1% wt., which made the concentrate too viscous to be used as a circulating fluid in heat exchange systems.

### 2.1. The mPCM Slurry

The test fluid samples were prepared by adding distilled and demineralised water to the concentrate (MICRONAL^®^ 5428 X) a product of Microtek Labs (Moraine, OH, USA). Seven slurries with different concentrations of PCM microcapsules were prepared by mechanical mixing of the components. They were 4.30%, 6.45%, 8.60%, 10.75%, 12.90%, 15.05% and 17.20% wt. The slurries prepared were homogeneous and characterised by the long-term stability of their physical properties (no visible signs of phase separation due to gravitational interactions). Each slurry was subjected to detailed tests to determine its physical properties precisely as a function of temperature. Among other things, the density of the slurry, its viscosity and specific enthalpy were determined. The measurements were carried out for several dozen temperature values ranging from 10 °C to 50 °C.

The procedure for determining the density of the mPCM slurry and sample test results was presented in the authors’ study [31]. The conducted tests showed that with increasing temperature, the density of the slurry decreased slightly. After reaching the phase transition temperature of the paraffin, the increase in its volume due to the transition from the solid to the liquid state resulted in a significant decrease in the density of the slurry. When the phase transition process of the PCM was completed, a further increase in the temperature of the slurry again resulted in a slight decrease in its density. A comparison of the test results and theoretical calculations of the thermal expansion of materials described in the paper [31] indicated that the material of the microcapsule shell (polymethyl methacrylate polymer) did not hinder the possibility of significant changes in the volume of the paraffin. The higher the concentration of microcapsules in the slurry, the lower its density, and the effect of the phase transition of the PCM on a significant reduction in viscosity was more pronounced. The detailed values of the density of the mPCM slurry were used during the calculations of the test results.

The authors’ studies [32,33,34,35] presented the results of slurry viscosity measurement as a function of temperature and shear rate. They show that the viscosity of the tested slurry asymptotically decreased with increasing shear rate. Moreover, when the slurry viscosity was higher than the viscosity of the base liquid (water), the concentration of microcapsules in the slurry was higher. The effect of temperature on the slurry viscosity was noticeable, i.e., the higher the slurry temperature, the lower its lightness. The results of the mPCM slurry viscosity tests were used in further analyses aimed at determining the critical Reynolds number. The authors’ own studies of the density and specific enthalpy [36] of slurries prepared for flow tests showed that PCM enclosed in microcapsules underwent a phase change in the temperature range from 22 °C to 27 °C.

### 2.2. Experimental Setup

**Figure 1:**
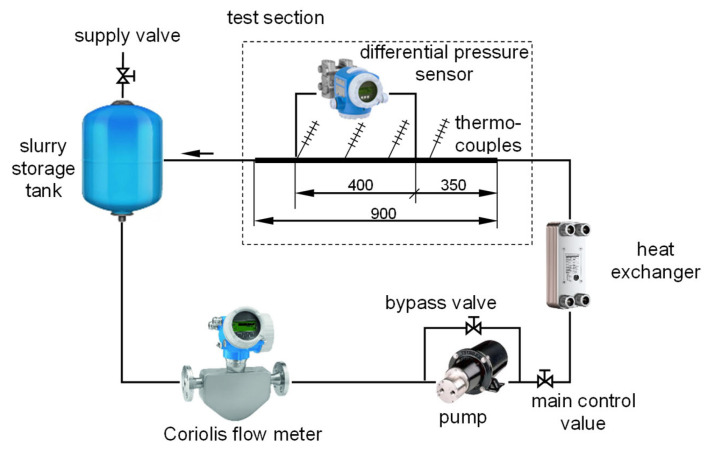
Pictorial scheme of the test stand [37].

Flow tests of mPCM slurries were carried out using an individually designed and constructed test stand. Its diagram is shown in Figure 1.

A miniature gear pump ensured the circulation of the working fluid (mPCM slurry) inside the installation. The flow rate of the slurry reaching the test section was regulated by changing the primary control value and bypass value settings. The measurement of the flow rate of the slurry circulating in the test circuit was possible with a Coriolis-type mass flow meter. This was a Promass 80 flow meter by Endress-Hauser (Wien, Austria). The mPCM slurry, with a known flow rate, reached the plate heat exchanger. By using an intermediate fluid flowing through the heat exchanger, it was possible to adjust the temperature of the slurry to the desired value. The slurry temperature was determined as the average value of the readings of four thermometers installed on the surface of the tube in the test section. The role of thermometers was played by individually made and marked K-type thermocouples. The temperature of the slurry was measured in the places shown in Figure 1. Since the entire test section was thermally insulated, the readings of the thermoelectric thermometers practically did not differ, and it could be assumed that the slurry temperature in the entire test section did not change.

The main element of the test section was a straight stainless steel tube with an internal diameter of d = 4 mm and a total length of 900 mm. The initial section of the tube (350 mm long) acted as a hydraulic stabilization zone for the flowing slurry. The next section of the tube (length L = 400 mm) acted as the main measuring section. The pressure drop caused by the flow resistance of the slurry through the tube was measured along this section. The flow resistance was measured using a piezoresistive differential pressure sensor: the Deltabar S PMD75 by Endress-Hauser (Wien, Austria). The last section of the tube, 150 mm long, acted as a stabiliser for the flow of the medium leaving the measuring section. The slurry leaving the test section flowed through the liquid reservoir to the flowmeter, closing the medium circuit. The uncertainty of the measuring equipment is presented in Table 1.

### 2.3. Research Procedure and Data Calculation

Reynolds, based on a series of his own experimental studies, defined the Reynolds number, the value of which is the basis for assessing the nature of fluid movement. Based on the observation of the behaviour of a coloured stream, he classified the flow of liquids into laminar flow and turbulent flow. The method has several disadvantages: (a) it is not suitable for observing the nature of movement in fluids that are not transparent (such as mPCM water suspensions), (b) it relies on a visual assessment of the dye stream path and a subjective assessment of the moment of its change, (c) it is very sensitive to any vibrations transferred from the environment and (d) it requires the use of channels with diameters that allow the introduction of a dye-feeding nozzle. A precise determination of the transition boundary from laminar to turbulent flow can be made based on the Poiseuille number, which is constant as long as the fluid flow is laminar. This approach requires numerous experiments conducted in a wide range of Reynolds numbers to be effective. The authors successfully used it in the studies described in the paper [38].

According to the results of Darcy–Weisbach tests, the pressure drop ∆p [Pa] of a liquid in a flow through a straight pipe of length L [m] with an internal diameter d [m] depends on the type of liquid (its density ρ (kg/m^3^)), the average liquid flow velocity in (m/s) and the linear loss factor (Darcy friction factor λ (−)), which can be expressed by the following relationship:

(1) $$\frac{∆p}{L}=\lambda \times \frac{1}{d}\times \frac{\rho w^{2}}{2}$$

On the other hand, according to the Hagen–Poiseuille law, when the fluid flow is laminar, the following relation is valid:

(2) Po=λ×Re=const

where Po (−) denotes the Poiseuille number, and Re (−) is the Reynolds number. The Poiseuille number is a constant value that depends on the channel shape (e.g., for channels with a circular cross-section, Po = 64). The Reynolds number is determined from the following relationship:

(3) $$Re=\frac{w\times d}{\nu }=\frac{\rho wd}{\mu }$$

where ν (m^2^/s) denotes the kinematic coefficient of fluid viscosity, and μ (Pa·s)—the fluid viscosity dynamic coefficient. After converting the dependence into the mass flow rate of the fluid $\dot{m}$ (kg/s),

(4) $$\dot{m}=\varrho \;w\frac{\pi d^{2}}{4}$$

then replacing the velocity w from Equation (4) into Equation (3) gives the formula for the Reynolds number in the following form:

(5) $$Re=\frac{4\dot{m}}{\pi \mu d}$$

If the below condition is met,

(6) $$\lambda \times Re=\left(\frac{\Delta p}{L}\frac{2d}{\varrho w^{2}}\right)\left(\frac{4\dot{m}}{\pi \mu d}\right)=0.5\pi \frac{\Delta p}{L}\frac{\rho d^{4}}{\dot{m}}=const$$

it can be assumed that the fluid flow is laminar. The value of the Reynolds number at which the Poiseuille number starts to deviate from the constant value should be considered the critical Reynolds number (Re_cr_). Taking the above into account, for each slurry flow rate m inside a tube of diameter d, the pressure drop ∆p should be measured over the section of length L.

## 3. Experimental Data

### 3.1. Pressure Drop in mPCM Slurry Flow

**Figure 2:**
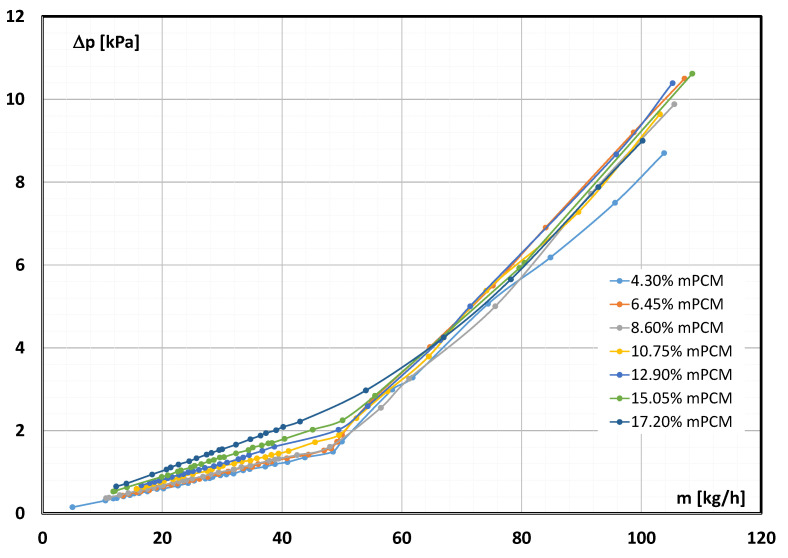
The mass flow rate effect of the mPCM slurry and its concentration on the pressure drop (slurry temperature T = 7 °C) [37].

Figure 2 shows sample results of the measured pressure drop caused by an mPCM slurry flow inside a tube with a diameter of d = 4 mm as a function of the mass flow rate. The pressure drop shown concerns a section of length L = 400 mm. The results presented in Figure 2 concern slurry testing at a temperature T = 7 °C and an mPCM concentration from 4.30% to 17.20%.

From Figure 2, it can be seen that for each slurry concentration, two different trends of the characteristic course can be distinguished. In the flow rate range up to about m = 50 (kg/h), the points illustrating the pressure drop were arranged along a straight line. The rectilinear course of the characteristic ∆p = f($\dot{m}$) indicates the laminar flow of the slurry. After exceeding this value, the course of the characteristics changed, which suggests a change like fluid movement. The higher the mPCM concentration, the slightly higher the flow rate at which the characteristic trend changed. A similar relationship can be seen in research work [19].

**Figure 3:**
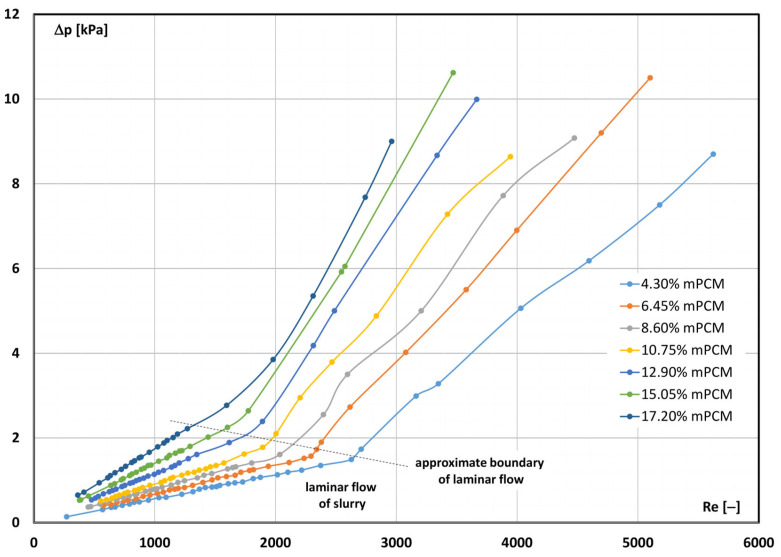
The Reynolds number and mPCM slurry concentration effect on the pressure drop (slurry temperature T = 7 °C) [37].

It is noted that with the increase in the concentration of microcapsules, a higher flow rate is required for the laminar flow to start changing into turbulent flow. The reason for such behaviour of the slurry is the increase in its viscosity. The higher the viscosity of the slurry, the lower the value of the Reynolds number corresponding to this flow, according to Formula (5). The authors of study [16] drew a similar conclusion. The effect of the Reynolds number on the pressure drop caused by the flow of the mPCM slurry inside the tube with a diameter of d = 4 mm is shown in Figure 3. The Reynolds number was calculated according to relationship (5). The pressure drop was measured on a section of length L = 400 mm during the flow of the slurry at a temperature of T = 7 °C and an mPCM concentration from 4.30% to 17.20%.

From Figure 3, it can be concluded that for each slurry concentration, two different trends of the course of the characteristic can be distinguished. The change in the trend of the characteristic takes place at a different value of the Reynolds number. It is also noted that the higher the concentration of mPCM in the slurry, the higher the measured values of the pressure drop during its flow. Moreover, the higher the slurry concentration, the more difficult it was to maintain the laminar flow of the slurry. As a result, the change, like the fluid movement, took place at a lower value of the critical Reynolds number.

The influence of the slurry concentration on the inability to maintain the laminar nature of the slurry movement can be explained by the frequency of collisions of microcapsules flowing in parallel layers of fluid. The higher the concentration of microcapsules, the greater the probability of their collision. As a result of collisions, microcapsules were knocked out of their track, moved from their layer to the parallel layer and knocked out other microcapsules and liquid particles from the ordered flow along the tube axis. As a result, at a lower Reynolds number, the entire fluid changed from a stratified flow to a turbulent flow.

**Figure 4:**
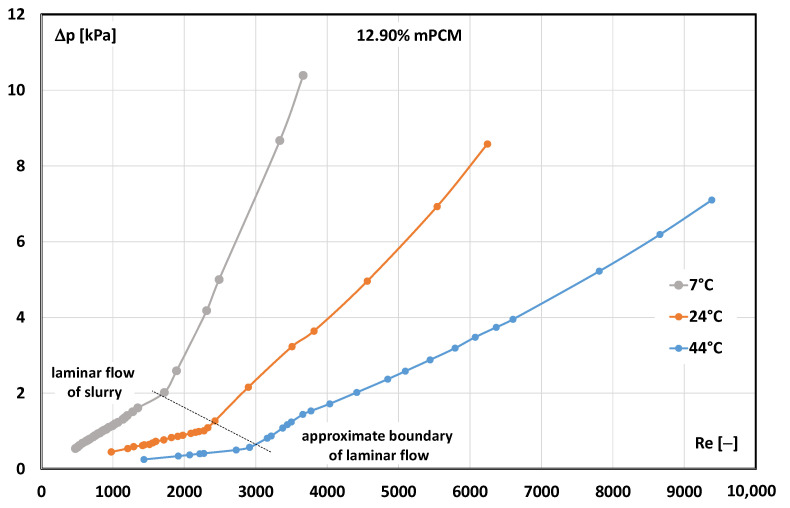
The influence of the Reynolds number and the temperature of the mPCM slurry on the pressure drop.

The effect of the slurry temperature on the flow resistance and the critical Reynolds number is shown in Figure 4.

Figure 4 shows that the fluid temperature significantly affected the pressure drop value during the slurry flow. The higher the slurry temperature, the lower the pressure drop value recorded at the test stand. The effect of the temperature increase on the decrease in flow resistance seems obvious. It results from the decrease in the viscosity of the flowing liquid, which is the effect of reducing the forces of interaction between molecules when the distance between them is more significant. The increase in the distance between molecules results from the thermal expansion of the liquid.

The effect of the slurry temperature on the critical Reynolds number is more interesting. This phenomenon is not observed in the case of “pure” liquids, and the critical Reynolds number in the flow of, e.g., water is not temperature-dependent. The experiments showed that the higher the temperature of the mPCM slurry, the higher the Reynolds number, and a fluid movement-like change occurred. According to the authors, this phenomenon can be explained by the type of microcapsule collisions depending on the state of aggregation of the paraffin inside the shell. When the paraffin was solid, the microcapsule collisions were springy. When the paraffin in the microcapsules was in a liquid state, the microcapsule collisions were plastic. Plastic collisions of microcapsules absorb the momentum of the particles, and the energy of the detached microcapsules is no longer as large as that of the microcapsules rebounded elastically. Hence, the tendency of the microcapsules with liquid paraffin to pass to the neighbouring layer and knock out the liquid particles and microcapsules from motion is smaller. As a result, the flow of the slurry with liquid paraffin in the microcapsule allows for longer maintenance of laminar motion.

### 3.2. Critical Reynolds Number

**Figure 5:**
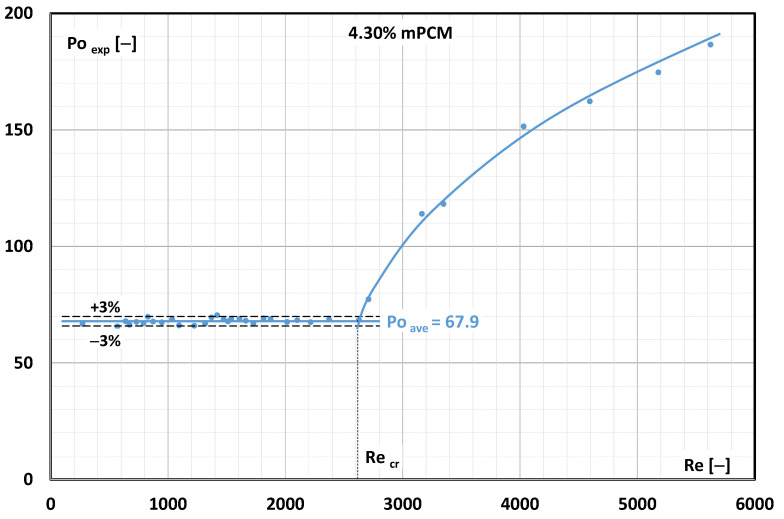
Effect of Reynolds number on experimental Poiseuille number (4.30% mPCM slurry, temperature T = 7 °C).

Using relationship (6), the Poiseuille number was calculated. The results of the calculations of the Poiseuille number value based on the collected experimental data (Po_exp_) from the Reynolds number are shown in Figure 5.

**Figure 6:**
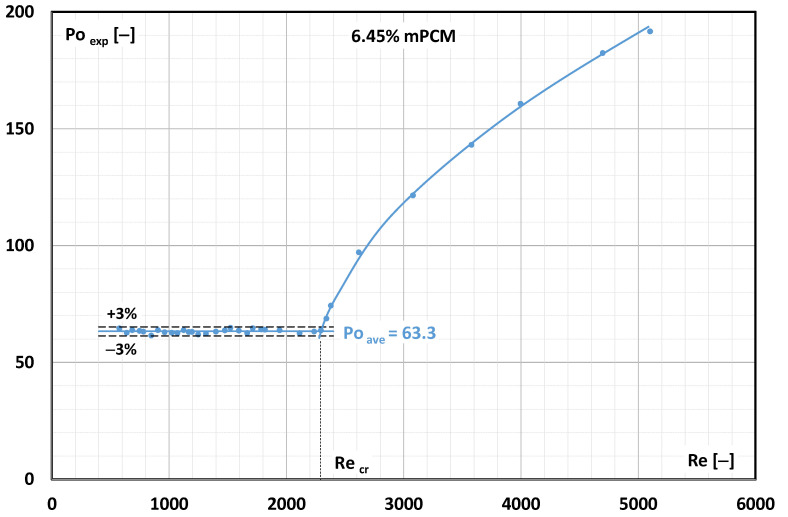
Effect of Reynolds number on experimental Poiseuille number (6.45% mPCM slurry, temperature T = 7 °C).

The calculation results presented in Figure 5 refer to experiments performed using a 4.30% mPCM water slurry at a temperature of T = 7 °C. It is known from fluid mechanics that laminar fluid flow occurs as long as the Po number is a constant value. For the presented case, the change in the nature of fluid motion (constant Po number) persisted up to Re = 2600. Hence, it was assumed that for the flow of the mPCM slurry at a concentration of 4.30% and a temperature of T = 7 °C, the critical Reynolds number was Re_cr_ = 2600. When the mPCM slurry concentration increased to 6.45%, the critical Reynolds number was already lower and amounted to approximately Re_cr_ = 2300 (Figure 6).

**Figure 7:**
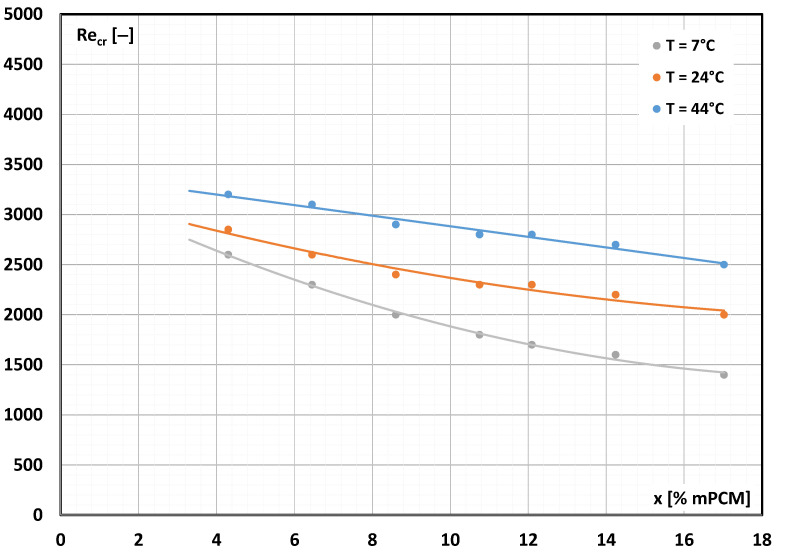
The mPCM slurry concentration and its temperature influence on the critical Reynolds number.

Based on the graphically determined value of the critical Reynolds number for each tested case, a summary was prepared, which is presented in Figure 7. The figure shows the effect of the mPCM slurry concentration and the slurry temperature (the state of matter of the PCM in the microcapsule) on the critical Reynolds number.

Extrapolation of the experimental results allows us to assume that if water without the addition of mPCM (x = 0% mPCM) were used in the test stand, the critical Reynolds number would be approximately Re_cr_ = 3500. This is a higher value than Re 2300, which is considered the engineering criterium for dividing flows into laminar and turbulent. It should be remembered that the value of Re = 2300 is the so-called lower critical Reynolds number. When the fluid flow rate increases, it is possible to maintain laminar fluid flow significantly above this value. It is noted that the increased concentration of the slurry made it more difficult to maintain its laminar flow. Additionally, when the paraffin enclosed in the microcapsule was a solid (T = 7 °C), the transition to turbulent flow occurred at a much lower Re_cr_ value than when the paraffin changed its state of matter. The highest values of the critical Reynolds number were obtained when microcapsules filled with liquid paraffin flowed in the liquid. These studies also show that the mPCM slurry changed the nature of the movement before the value of Re = 2300 was reached.

These studies show that the concentration of microcapsules and the temperature of the mPCM suspension affect the value of the critical Reynolds number. It is noted that when the PCM in the microcapsule was solid, a departure from laminar fluid flow took place at a value of Re much lower than Re = 2300 (already at Re = 1500 for 17.20% PCM). Therefore, the theoretical formulas for calculating the laminar heat transfer coefficient and laminar flow resistance, in the case of 17.2% mPCM suspension, lose their validity after exceeding Re = 1500. Therefore, the intensification of heat transfer and turbulence of the mPCM slurry flow occur much faster than expected from the current state of knowledge.

## 4. Conclusions and Summary

Experimental flow studies were conducted to determine the effect of the concentration of microcapsules containing paraffin and the state of aggregation of paraffin on the critical Reynolds number. The studies were carried out using seven slurries with different concentrations of PCM microcapsules. They were 4.30%, 6.45%, 8.60%, 10.75%, 12.90%, 15.05% and 17.20% wt. The experiments were conducted in a wide range of Reynolds numbers—up to Re = 11,250. The slurry temperature during the studies was constant and amounted to 7 °C, 24 °C and 44 °C. Such a value of the slurry temperature caused the PCM in the microcapsule to become a solid, undergo a phase change and become a liquid. To determine the critical Reynolds number, the Poiseuille approach was used, according to which the Poiseuille number assumes a constant value for laminar, incompressible and steady fluid flow in rectilinear channels of constant diameter. The Poiseuille number is the product of the friction coefficient and the Reynolds number. Hence, the research was carried out based on the precise determination of the pressure drop in the flow of the mPCM slurry and its properties as a function of temperature. The obtained research results and the conducted analyses show the following:

- Adding microcapsules containing PCM to the base liquid (water) affects the critical Reynolds number.
- The higher the concentration of mPCM in the slurry, the more difficult it was to maintain laminar movement.
- The transition from laminar to turbulent movement occurred at Re < 2300 (e.g., already at Re ≈ 1400 for 17.20% mPCM slurry at temperature T = 7 °C).
- Tensile interactions between capsules filled with solid paraffin meant that the transition to turbulent flow occurred at a much lower Reynolds number than when the slurry contained microcapsules with liquid paraffin.

Further studies are planned to precisely determine the effect of slurry temperature (PCM state of matter) on the critical Reynolds number. The common assumption is that the Re_cr_ value for the mPCM slurry flow is the same as for the base fluid (water) flow and that the presented experimental results did not confirm Re_cr_ = 2300.

## Figures and Tables

**Table 1:** The uncertainty of the measuring equipment.

Equipment	Range	Uncertainty
Mass flow meter	0–110 kg/h	±0.2% of the measured value
Differential pressure sensor	0–50 kPa	±0.075% of the maximum value (±37.5 Pa)
K-type thermocouples	−40 °C–+475 °C	±0.2 K

## Data Availability

The original contributions presented in this study are included in the article. Further inquiries can be directed to the corresponding author.
